# DPP-4 Inhibitors as a savior for COVID-19 patients with diabetes

**DOI:** 10.2217/fvl-2022-0112

**Published:** 2023-04-11

**Authors:** Snehasish Nag, Samanwita Mandal, Oindrila Mukherjee, Suprabhat Mukherjee, Rakesh Kundu

**Affiliations:** ^1^Department of Zoology, Cell Signaling Laboratory, Visva-Bharati University, Santiniketan, West Bengal, 731 235, India; ^2^Department of Animal Science, Integrative Biochemistry & Immunology Laboratory, Kazi Nazrul University, Asansol, West Bengal, 713 340, India

**Keywords:** cytokine storm, diabetes, dipeptidyl peptidase-4 (DPP-4), gliptins, SARS-CoV-2, TLR4 signaling

## Abstract

Diabetic patients are at particular risk of severe COVID-19. Human dipeptidyl peptidase-4 (DPP-4) is a membrane-bound aminopeptidase that regulates insulin release by inactivating incretin. DPP-4 inhibitors (DPP-4is) are therefore used as oral anti-diabetic drugs to restore normal insulin levels. These molecules also have anti-inflammatory and anti-hypertension effects. Recent studies on the interactions of SARS-CoV-2 spike glycoprotein and DPP-4 predict a possible entry route for SARS-CoV-2. Therefore, DPP-4is could be effective at reducing the virus-induced ‘cytokine storm’, thereby ceasing inflammatory injury to vital organs. Moreover, DPP-4is may interfere with viral entry into host cells. Herein, we have reviewed the efficacy of DPP-4is as potential repurposed drugs to reduce the severity of SARS-CoV-2 infection in patients with diabetes.

## Tweetable abstract

DPP-4 inhibitors could be promising repurposed drugs to minimize inflammatory pathogenesis and disease severity in COVID-19/diabetes comorbidity.

A pandemic caused by a beta class of coronavirus called SARS-CoV-2 has engulfed the world since its origin in December, 2019 [1]. According to the WHO, the virus has already infected more than 662 million people and has caused over 6 million deaths worldwide as of 16 January 2023 [2]. Preliminary reports suggest an increased risk of severe disease in patients with diabetes. Around 15% of critically ill COVID-19 patients have diabetes, and the rate of mortality is two to three-times higher compared with non-diabetic patients [3]. Such a high mortality among COVID-infected diabetes patients requires urgent therapeutic intervention. Early findings suggest a potential role of anti-diabetic drugs, including dipeptidyl peptidase-4 (DPP-4) inhibitors. DPP-4 inhibitor drugs, called gliptins, neutralize the proteolytic action of DPP-4 on incretin hormones glucagon-like peptide-1 (GLP-1) and gastric inhibitory polypeptide (GIP) preventing the reduction in insulin secretion [4]. By inhibiting DPP-4, gliptins also reduce the production of pro-inflammatory cytokines and the CRP level, which may protect the lung from inflammatory injury during novel coronavirus infection [5,6]. DPP-4 inhibitor Sitagliptin inhibits Toll-like receptor 4 (TLR4) activation in alveolar cells in the lung. Given that the SARS-CoV-2 surface antigen spike glycoprotein interacts with TLR4 and induces inflammatory cascades, leading to ‘cytokine storm’, it has been suggested that DPP-4 inhibitors may prevent virus-TLR4 interaction and the consequences of multi-organ failure [7,8]. It is well established that the entry of SARS-CoV-2 into host cells is through binding its cognate receptor, ACE2 [9]. However, during MERS-CoV (Middle East respiratory syndrome coronavirus) infection, DPP-4 has emerged to be the major entry receptor utilised by the virus to infect host cells. Thus, it is possible that the SARS-CoV-2 spike glycoprotein may also interact with DPP-4 [10,11]. Available literature suggests that membrane-bound DPP-4 may promote the entry of SARS-CoV-2 into the respiratory system [12,13]. A recent clinical study showed that COVID-19 patients had reduced levels of soluble DPP-4 (sDPP-4), possibly due to reduced shedding of the catalytic domain from its transmembrane domain compared with the uninfected healthy subjects. Therefore, the high amount of intact membrane-bound DPP-4 in infected patients may contribute to severity of the disease [12]. This suggests that repurposing DPP-4 inhibitors could be effective for treating COVID-19 patients with diabetes.

## DPP-4 & its implications in COVID-19

DPP-4 is a ubiquitous proteolytic enzyme that cleaves and inactivates several biologically active substrates such as GLP-1 of the incretin pathway, leading to reduced insulin secretion from pancreatic beta cells and thus resulting in the diabetic condition [4]. DPP-4, or cluster of differentiation 26 (CD26), is a membrane-bound serine ectopeptidase that shows catalytic activities by cleaving the N-terminus of dipeptides with a proline amino acid in its penultimate position [14]. DPP-4, through its intracellular tail, transmits signals by cleaving proline, alanine and glycine [15]. N-terminal sialylation of membrane-bound DPP-4 increases its apical secretion, and thus may promote virus interaction [16], as is demonstrated in the case of MERS-CoV, where DPP-4 interacts with the N-glycan-containing receptor-binding domain (RBD) of the virus [17,18]. The membrane-bound form of DPP-4 is ubiquitous, present in T lymphocytes, monocytes, hepatocytes, kidney, lung, small intestine, pancreas, spleen and the heart [14,19,20]. The lung is rich in DPP-4 and is the site most affected by novel coronavirus. The role of DPP-4 expression in the severity of coronavirus infections is already demonstrated in the case of MERS-CoV, where increased level of DPP-4 in the lung leads to inflammation by acting as a pro-inflammatory signaling molecule [21].

In addition to the membrane-bound form, DPP-4 is present as a soluble form in plasma and other body fluids [22,23]. The enzymatically-active sDPP-4 is cleaved from the membrane-bound form and released. Some clinical studies have observed increased levels of sDPP-4 in the plasma of patients suffering from viral infections [24]. However, in patients with MERS-CoV, a reduced level of sDPP-4 correlated with disease severity [13]. A similar relationship has also been found in the case of COVID-19 infection [12,25]. An earlier study also found that levels of sDPP-4 were significantly higher in younger compared with older individuals [26]. This suggests a probable role of sDPP-4, in addition to membrane-bound DPP-4, in COVID-19 severity.

## Virus-DPP-4 interaction

Although ACE2 seems to be the principal receptor for SARS-CoV-2 entry, studies suggest that DPP-4 also interacts with the novel coronavirus [10,27,28]. Recent reviews and *in silico* analyses propose that several receptors such as DPP-4, DPP-6, DPP-10, TLR4, C-lectin type receptors (CLRs), neuropilin-1 (NRP1) may interact with the SARS-CoV-2 receptor-binding domain [11,29,30].

**Figure 1.**
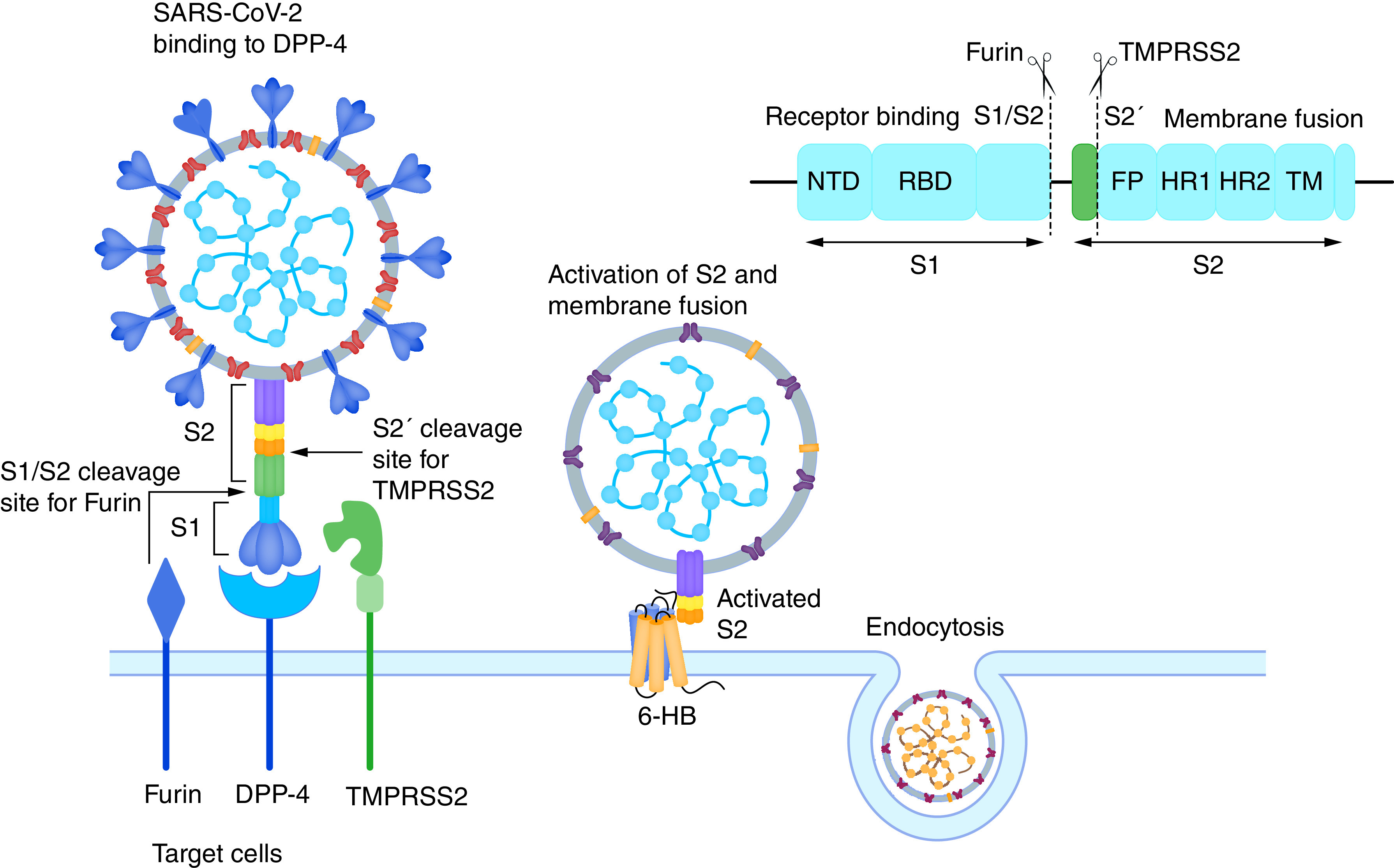
Entry of SARS-CoV-2 in host cell using DPP-4. Furin and TMPRSS2 are host cell proteases that cleave specific target regions in virus spike glycoprotein and promote endocytosis into host cells. Domain structure of SARS-CoV-2 spike glycoprotein with furin and TMPRSS2 cleavage sites are also shown.

Earlier studies with MERS-CoV suggested that membrane-bound DPP-4 acts as a functional receptor and mediates internalization of the virus into host cells. A recent publication using a homology modelling approach showed that the human membrane-bound DPP-4 interacts with receptor binding domain of the SARS-CoV spike glycoprotein, the S1 subunit. Many unique residues present on the S1 subunit showed interaction sites similar to the ACE2 receptor, indicating that membrane-bound DPP-4 could act as an entry receptor of SARS-CoV-2 in humans [10]. During entry of the host cell, the SARS-CoV spike glycoprotein is cleaved at the site of the S1-S2 junction by a membrane-bound protease, furin, releasing the S1 subunit. This then binds DPP-4 (Figure 1). Binding and cleavage exposes the S2 subunit, the membrane-fusion subunit, which is then cleaved at an S2’ site located upstream of the fusion peptide (FP) by another protease, transmembrane serine protease 2 (TMPRSS2). As a result, the S2' site of the viral spike protein is activated and forms a 6-helix bundle fusion core (6-HB) of heptad repeat 1 (HR1) and 2 (HR2) from S2 subunit. This promotes fusion of the host cell and viral membrane, and subsequent endocytosis of the virus (Figure 1) [31–34].

**Figure 2.**
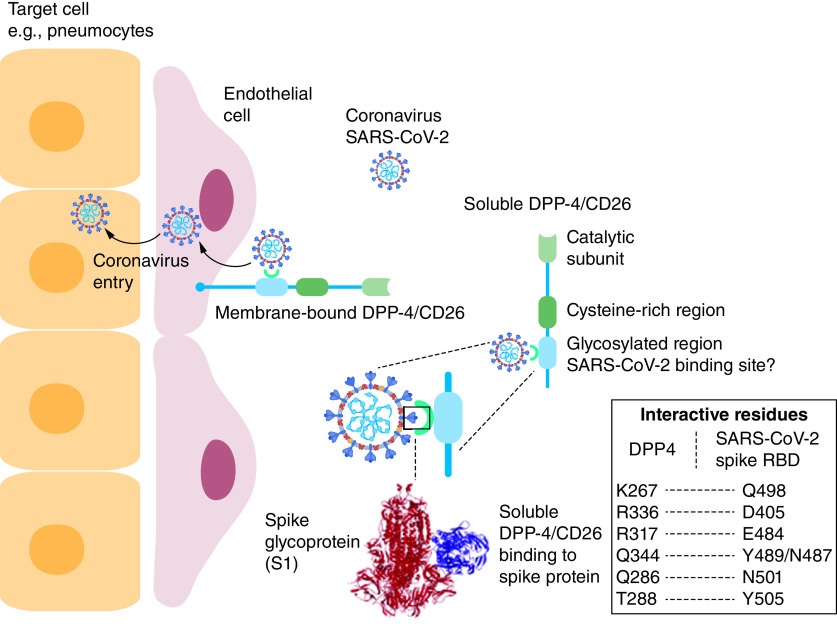
Hypothetical illustration of how SARS-CoV-2 interacts with membrane-bound DPP-4/CD26 to facilitate the entry of the virus into the host cells, for example the epithelial cells of alveoli. Viral spike glycoprotein can bind to both soluble and membrane-bound receptor. Binding of spike protein to membrane-bound DPP-4 facilitates the viral entry to the target cell. The exact domain of DPP-4 wherein the spike protein occupies, is an open question. Potential interactive residues between DPP-4 and SARS-CoV-2 spike RBD have been shown [11].

Competitive inhibition studies revealed that sDPP-4 acts as decoy receptor and blocks the internalization of the virus [35]. Soluble DPP-4 at a dose of 250 and 1000 ng/ml inhibited the entry of the virus by 14 and 24% respectively. To reach 50% inhibition of entry, a concentration of at least 8000 ng/ml soluble DPP-4 is needed [13]. One hypothesis is that the viral spike protein may interact with the non-catalytic glycosylated region of DPP-4, which facilitates SARS-CoV-2 entry and thus virulence in host cells. Clear correlations between ACE2 and DPP-4 suggests the relevance of both of these membrane-bound proteins in the pathogenesis of SARS-CoV-2. Preliminary reports suggest that, as in MERS-CoV infection, there is a correlation between the localization of membrane-bound DPP-4 and the site of lung inflammation in SARS-CoV-2, supporting the interactions between DPP-4 and the virus [21]. Although some studies suggest that ACE2 is the major binding partner of SARS-CoV-2, rather than DPP-4 as is the case for MERS-CoV, this does not rule out DPP-4 playing a role in the internalization of SARS-CoV-2. For example, patients with Type 2 diabetes have shown increased risk for the COVID-19 [12,36]; Schlicht *et al.* performed a clinical study where they measured the circulating level of sDPP-4 in 7 patients suffering with COVID-19 disease and found the level of sDPP-4 to be significantly reduced in these patients in comparison to healthy control [12]. This may suggest that reduced shedding of membrane-bound DPP-4 leads to increased likelihood of SARS-CoV-2 internalization as it acts as a receptor for the viral spike protein. The mechanistic insights of host-virus interaction, mediated by soluble and membrane-bound DPP-4, have been summarized in Figure 2.

Like other RNA viruses such as SARS-CoV and MERS-CoV, it is evident that SARS-CoV-2 shows a high rate of mutation, adapting to environmental changes and pathogenic selection pressures. Interaction of this rapidly mutating virus with DPP-4 in conjunction with ACE2 presents the possibility of using DPP-4 as the principal entry receptor in the future [37].

## DPP-4 inhibitors & COVID-19

DPP-4 inhibitors, known as Gliptins, are a class of oral anti-diabetes drugs. Commercially available DPP-4 inhibitors include Galvus (Vildagliptin), Januvia (Sitagliptin), Onglyza (Saxagliptin) and Tradjenta (Linagliptin) [38]. DPP-4 inhibitors target the proteolytic enzyme DPP-4 and prevent inactivation of the incretin pathway leading to increased insulin secretion [39].

As preliminary clinical data suggests that membrane-bound DPP-4 plays an important role in SARS-CoV-2 virus entry into the human respiratory system [12], this suggests a possible role for anti-diabetic DPP-4 inhibitors in reducing the severity of SARS-CoV-2 infection [40,41]. Currently, very few studies have been performed with patients taking DPP-4 inhibitors for COVID-19 [37]. One study observed that the mortality of patients with a well-controlled blood glucose level was significantly lower than that of patients with poorly-controlled blood glucose levels; it was found that the majority of patients with well-controlled blood glucose levels were receiving DPP-4 inhibitors as anti-diabetic drugs [42]. Although this report does not prove that DPP-4 inhibitors directly reduce the severity of COVID-19, it presents a possible treatment strategy worth investigating further. A few studies performed in diabetic patients have shown that no significant changes were observed in DPP-4 inhibitor users [43–45]. This at least suggests that anti-diabetic DPP-4 inhibitor drugs are not harmful in COVID-19 patients. At least one study, however, has shown that patients taking DPP-4 inhibitors required less intubation (43%) than non-users (81%), suggesting a beneficial role of DPP-4 inhibitors in COVID-19 [46]. Another population-based study showed that out of 832 diabetic patients with novel coronavirus disease, the 263 patients who were taking DPP-4 inhibitors showed 64% less severe symptoms of COVID-19 than non-users [47]. DPP-4 inhibitors, when administered in the inpatient setting, also seem to reduce the risk of COVID-19-associated deaths by about 50% [48]. Currently, several studies using DPP-4 inhibitors such as sitagliptin and linagliptin are in progress to investigate the beneficial effects on COVID-19 disease [21]. Preliminary reports on the beneficial effects of DPP-4 inhibitors on COVID-19 are, so far, promising.

## Repurposing DPP-4 inhibitors in COVID-19 treatment

Studies suggest that SARS-CoV-2 infects the lower airways and can cause acute respiratory distress syndrome (ARDS) [49,50]. Cytokine storms are also produced as result of SARS-CoV-2 infection [8]. Cell entry of SARS-CoV-2 is at least partly regulated by the membrane-bound DPP-4 [12]. These studies, taken together, indicate the potential role of DPP-4 inhibitors in antagonizing the effects of the virus [21,51,52]. Continued use of DPP4 inhibitors in hospitalized patients have been found to reduce mortality of COVID-19 patients [53,54]. DPP-4 inhibitors prevent airway inflammation [55] and thus their use may reduce the virulence of SARS-CoV-2 and the resulting multi-organ damage. Possible mechanisms of action include: interaction of the virus with CD26 [12]; reduced cytokine production [56]; reduced macrophage activity [57]; increased GLP-1 activity [58]; and stimulation of anti-inflammatory effects of the lungs and other tissues [7,59,60]. Thus, DPP-4 inhibitors may be most effective when administered in the early phases of SARS-CoV-2 infection, and so could then be used in combination with other treatments to reduce the severity of resulting COVID-19 disease.

DPP-4 inhibitors supress the proliferation of T cells. This results in the reduced production of pro-inflammatory cytokines and thus reduces the ‘cytokine storm’. This is evident in studies where patients taking DPP-4 inhibitors have shown 30% lower occurrence of auto-immune diseases [61]. However, caution must be taken as suppression of T cell immunity by DPP-4 inhibitors may increase the risk or severity of acute SARS-CoV-2 infection [62].

DPP-4 inhibitor drugs seem to improve lung health in multiple ways. In the case of ARDS, the DPP-4 inhibitor sitagliptin inhibits production of pro-inflammatory cytokines such as IL-1β, IL-6 and TNF-alpha, which lessens lung injury [7]. It also seems to inhibit TGF-beta mediated lung fibrosis [63]. Using a mouse model, it was found that the DPP-4 inhibitor drug vildagliptin also induces natural killer (NK) cell activity, which leads to reduced lung cancer growth [64]. Although, few studies suggest that vildagliptin induces pneumonitis, which may increase the chances of fibrotic lesions in the lungs during SARS-CoV-2 infection and increase the severity of COVID-19 disease [65]. DPP-4 inhibition is also associated with some degree of immune suppression, and long-term suppression of the immune system could lead to undesirable side effects [66]. Thus, it has been suggested that inhibition of DPP-4 may not be the most desirable approach to reduce the severity of COVID-19 [67].

Several reports suggest regulatory effects of DPP-4 inhibitors on both systolic and diastolic blood pressure (SBP and DBP). Treatment with DPP-4 inhibitors such as sitagliptin, vildagliptin and saxagliptin appear to reduce SBP and DBP and protect patients against effects of hypertension [68]. This phenomenon seems to not be limited to only diabetic patients, as sitagliptin has been shown to also reduce blood pressure in non-diabetic patients [69]. Results from a retrospective cohort study performed in China suggests increased SBP and unstable SBP/DBP are associated with increased risk of intensive care unit (ICU) admission, heart failure and mortality, while hypertension appears to be a common comorbidity in COVID-19 patients [70]. The role of DPP-4 inhibitors in reducing blood pressure and hypertension further suggest its potential as a repurposed drug for treating COVID-19.

As DPP-4 is found both in membrane-bound and soluble form, recent reports suggest that the use of DPP4 inhibitors in diabetic patients significantly upregulates the circulatory levels of sDPP-4 [71]. sDPP-4 acts as a decoy receptor to the virus [13] and therefore the use of DPP-4 inhibitors may prevent interaction of SARS-CoV-2 with the membrane-bound DPP-4 by increasing the titre of the circulatory sDPP-4 level [35].

Studies using pharmacophore modelling and molecular docking have shown that peptidomimetic DPP-4 inhibitors exhibit highly specific binding to the SARS-CoV-2 viral protease. Out of the 12 DPP-4 inhibitors studied, 3 inhibitors (gemigliptin, linagliptin, evogliptin) showed the best ability to bind the viral protease. However, non-peptidomimetic DPP-4 inhibitors showed the least binding ability [72]. However, it remains to be understood whether the conformational changes induced by DPP-4 inhibitors can modify the interaction of DPP-4 with SARS-CoV-2 [37]. In an earlier *in vitro* study, it was found that infection of human coronavirus-Erasmus Medical Center (hCoV-EMC) on Huh-7 cells and primary human bronchial epithelial cells was significantly inhibited upon treatment with antibodies against DPP-4 [73]. However, treatment with DPP-4 inhibitors such as vildagliptin, sitagliptin and saxagliptin did not inhibit hCoV-EMC infection, which suggests that even after binding of these inhibitors, DPP-4 is unable to alter the substrate. However, an anti-DPP-4 monoclonal antibody (YS110) has been able to significantly supress MERS-CoV infection by targeting the site between the binding domain of the virus and the receptor [74].

A recent study proposed that DPP-4 amplifies SARS-CoV-2 infection in diabetic patients, and thus DPP-4 inhibitors are thought to be able to reduce the virulence of SARS-CoV-2 [27]. A serine protease inhibitor called camostat mesylate has been able to supress the infection of SARS-CoV-2 efficiently by inhibiting the TMPRSS2 protein, which if not inhibited, cleaves the spike proteins of the virus and allows its fusion and entry in to cells [36]. DPP-4 inhibitors inhibit DPP-4, itself a serine protease, and so there is a possibility that DPP-4 inhibitors could be repurposed for COVID-19 [37]. Since DPP-4 inhibitors also have anti-inflammatory properties. DPP-4 inhibitors such as saxagliptin reduce NLR family pyrin domain containing 3 (NLRP3) inflammasome activation and also serum levels of TNF-alpha, IL-1β, IL-6 and CRP in diabetic mice models [5]. In a human study, DPP-4 inhibitor sitagliptin also reduces the serum levels of CRP, IL-6 and *cd26* mRNA in mononuclear cells [75]. The role of DPP-4 inhibitors in reducing cytokine storms may alleviate the severity of COVID-19. Interestingly CRP levels in 86% of COVID-19 patients are found to be highly elevated [6].

Because DPP-4 inhibitors can suppress immunity by altering the T cells activity [62], this has led to some suggestions that DPP-4 inhibitors might increase the risk of infections such as nasopharyngitis and interstitial pneumonitis in patients [65]. However meta-analyses and also large-scale patient studies performed by Clinical Practice Research Datalink of United Kingdom (CPRD) found no such increased risk of infections in DPP-4 inhibitor users in comparison to other anti-diabetic drugs [76]. As preliminary data suggests an increased severity of COVID-19 in elderly patients with high levels of DPP-4 due to diabetic or obesity conditions, DPP-4 inhibitors may be repurposed as drugs for regulating SARS-CoV-2 infection.

## Dual potential of DPP-4 inhibitors against SARS-CoV-2 immunopathogenesis

The pathophysiology of COVID-19 is the cumulative result of virus-induced physiological changes (alteration in respiratory gas exchange and dysregulation of blood pressure) and the ‘cytokine storm’. Out of all the pathophysiological attributes, the cytokine storm is widely accepted as the prime cause behind COVID-19-related mortality. Like other host-pathogen interactions, SARS-CoV-2 infection is sensed via the cell surface and intracellular TLRs [8,77]. Both cell surface and intracellular TLRs have been implicated in COVID-19 immunopathogenesis. Intriguingly, the cell surface viral antigen – the spike glycoprotein – has been found to bind with TLR4, inducing the inflammatory cascade that is the most likely cause of the cytokine storm [8]. Therefore, targeted inhibition of TLR4 has already emerged as an effective treatment option, especially in patients displaying severe immunopathological manifestations [78]. A number of pharmacological agents (HCQ; phase III, Imiquimod; phase I), small molecules (PUL-042; phase III) and peptides (EC-18) are currently undergoing clinical trials [78].

**Figure 3.**
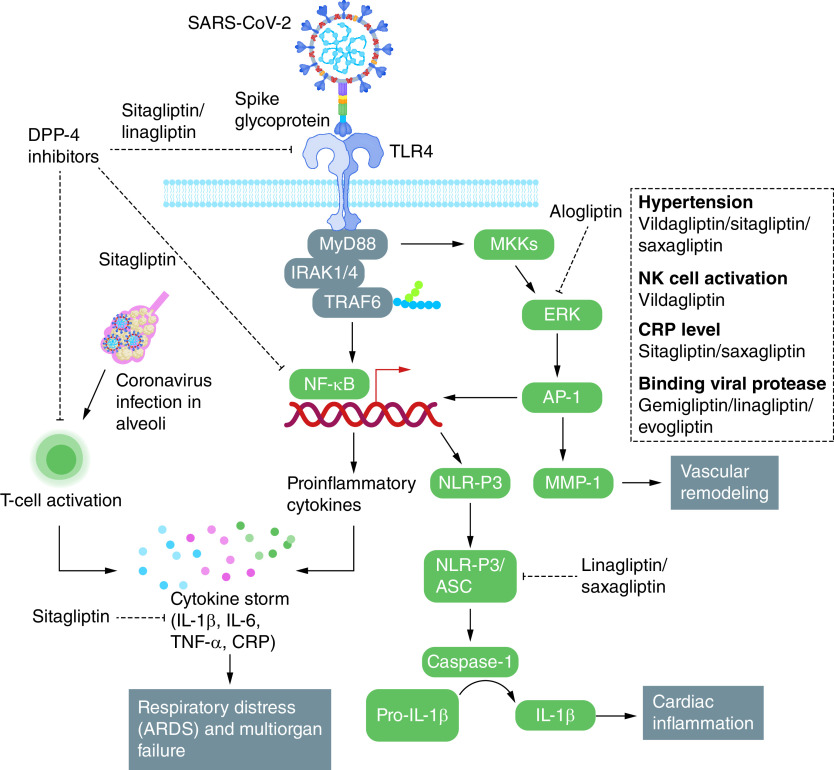
DPP-4 inhibitors targeting TLR4 mediated immunopathogenesis of COVID-19. DPP-4 inhibitors mediated therapeutic strategies to target human TLR4, NF-κB, ERK, NLR-P3/ASC and T-cell activation against SARS-CoV-2 and to protect multiorgan damage. The anti-DPP-4 drug sitagliptin can directly binds to TLR4 to inhibit the downstream inflammatory cascade while alogliptin inhibits ERK which is one of crucial the downstream signaling mediator originated of TLR4 signaling pathway. In direct inhibitory effects of the gliptins on the T cell-induced secretion of the pro-inflammatory cytokines has also been presented. These inhibitory actions could be useful in mitigating the cytokine storm in the COVID-19 patients. Inhibition of ERK plays useful role in limiting the effects of the MMPs on the blood vessels and also blocks the activation of AP-1 mediated synthesis of the inflammatory molecules. Linagliptin has inhibitory effects on the NLRP3-mediated activation of IL-1β which in turn prevents cardiac inflammation in COVID-19 patients.

In this context, currently available FDA-approved DPP-4 inhibitors appear to be the best candidates. These repurposed drugs could be considered as effective TLR4 antagonists for COVID patients experiencing a cytokine storm, especially given that DPP-4 is expressed in almost all the major immune cells (macrophages, dendritic cells, T cells, B cells, NK cells) contributing to COVID-19 immunopathology, especially the cytokine storm [79]. Among the various DPP-4 inhibitors studied so far, alogliptin has been documented for its efficacy in inhibiting TLR4-mediated extracellular-signal-regulated kinase (ERK) activation and ERK-dependent matrix metalloproteinase (MMP-1) expression by monocytes [80]. Sitagliptin, on the other hand, is known to prevent lung injury through inhibiting TLR4 activation [7] and could be useful in dampening the SARS-CoV-2-TLR4 interaction and the inflammatory consequences associated with this. Sitagliptin can selectively reduce the level of proinflammatory cytokines in COVID-19 patients through blocking NF-κB signaling [81]. The anti-inflammatory nature of DPP-4 inhibitors could also be useful in mitigating post-COVID cardiac inflammation, its pathological manifestations (arrythmias, acute coronary syndrome) and heart failure [82]. In fact, a major percentage of death in COVID patients and recovered patients has been found to be due to cardiac hyperinflammation, wherein TLR4 and NLRP3 activation were found as major immunopathological hallmarks [82,83]. Interestingly, linagliptin is known to ameliorate cardiac inflammation and dysfunction through blocking the NLRP3/ASC inflammasome [84]. Moreover, linagliptin has been demonstrated as inhibiting TLR4 activation [85]. Linagliptin, therefore, also has potential to ameliorate cardiac inflammation in COVID patients. Sitagliptin, alogliptin, vildagliptin, saxagliptin and linagliptin have shown promise in ameliorating the immunopathogenesis, cytokine storm and organ damage that result from SARS-CoV-2 infection [82,86]. An overview of the molecular targets of different DPP-4 inhibitors to mitigate the immunopathological consequences of SARS-CoV-2 routed through TLR4 and the associated signaling cascades has been presented in Figure 3. A particular benefit of repurposing DPP-4 inhibitors is that they are well characterized and approved in most countries. Clinical trials have started to validate the findings reported from the different parts of the globe claiming anti-DPP-4 drugs as therapeutics against COVID-19.

## Randomized trials

To evaluate the effects of DPP-4 inhibitors on COVID-19 patients in the context of diabetes, few randomized control trials are in progress [37]. Various clinical trials and case studies investigating the role DPP-4 inhibitors as possible therapeutics against COVID-19 are summarized in Table 1. In one such trial (NCT04542213), scientists selected about 70 COVID-19 patients with hyperglycemia and divided into two groups. Group 1 received insulin with 5 mg per day linagliptin drug while group 2 received only insulin [87]. It was found that among the group receiving linagliptin, only 3 required assisted mechanical ventilation and there were 2 deaths, compared with 12 patients needing mechanical ventilation and 6 deaths in the group that only received insulin. This suggests a positive effect of the DPP-4 inhibitor on SARS-CoV-2 severity. Linagliptin treatment also improved pre- and post-prandial glucose levels in SARS-CoV-2 infected patients [88]. In another trial (NCT04371978), scientists used the DPP-4 inhibitor linagliptin at a dose of 5 mg per day on 32 patients suffering with diabetes and COVID-19. Their objective was to see whether linagliptin can cause any clinical improvements within 28 days [89]. They observed clinical improvement in 7 days, with 5 deaths in linagliptin-treated patients, compared with clinical improvement in 8 days with 8 deaths in non-treated patients [90]. This trial suggested that although DPP-4 inhibitor did not significantly improve the clinical conditions of COVID-19 patients with diabetes, the inhibitor also did not have any negative effect on their condition. In a further trial, 89 COVID-19 patients were recruited and divided into two groups. Group A received standard therapy (n = 40) while Group B received sitagliptin in addition to standard therapy (n = 49) for 28 days. Complete blood count (CBC), CRP, D-dimer, lactate dehydrogenase (LDH) and serum ferritin levels were measured for COVID-19 severity and complications [91]. The findings of this study suggested that sitagliptin in addition to standard therapy improved clinical outcomes in non-diabetic COVID-19 patients, when compared with only standard therapy. A separate trial by the same group also found positive effects of sitagliptin in combination with metformin and standard therapy in T2DM patients with COVID-19 [92]. More randomized and real-world trials are necessary to reach any conclusion regarding the role of DPP-4 inhibitors in regulating COVID-19.

**Table 1.** Clinical trials supporting the role of DPP-4 and its inhibitors in combatting COVID-19 patients with diabetes.

Trial category	Goal(s)	Major findings	Ref.
Case–control study	Assessing the concentration of sDPP-4 in the serum of COVID-19 patients	sDPP-4 level was significantly reduced in COVID-19 patients compared with healthy controls	[12]
Retrospective studies	Association between blood glucose level and the clinical outcomes of COVID-19 in Type 2 diabetic patients	Majority of DPP-4 inhibitor users displayed lower blood glucose levels which significantly reduced COVID-19 related mortalities	[42]
	Clinical outcomes of patients suffering with both diabetes and COVID-19 and effects of glucose lowering drugs (Metformin, DPP-4 inhibitors)	Clinical outcomes of DPP-4 inhibitor users were similar to non-users and the drugs found to have no association with in-hospital deaths	[43]
	Calculated the number of DPP-4 inhibitor users among COVID-19 patients having type 2 diabetes	Percentage of COVID-19 patients without taking DPP-4 inhibitors (82.9%) appeared similar to patients taking DPP-4 inhibitors (88.9%), suggesting no positive effects of the drug	[44]
Case series study	Rate of intubation between DPP-4 inhibitor takers and non-takers were studied among COVID-19 patients with diabetes and/or hyperglycemia	Diabetic patients with COVID-19 not using DPP-4 inhibitors showed much more intubation (81%) as compared with DPP-4 inhibitor users (43%)	[46]
Population-based study	Patients suffering with both COVID-19 and diabetes were studied to see the effects of DPP-4 inhibitors on disease severity leading to Intensive Care Unit (ICU) admission and death	Patients using DPP-4 inhibitors showed much less severity for COVID-19 with ICU admissions and deaths (3.42%) as compared with the non-users (4.39%)	[47]
Interventional, Clinical Trials	Approximately 64 COVID-19 patients with diabetes were given Linagliptin orally at 5mg/day to study the efficacy and safety of the drug	32 Patients receiving Linagliptin showed clinical improvement in 7 days with 5 deaths compared with clinical improvement in 8 days with 8 deaths in 32 non-treated patients	[89,90] (NCT04371978)
	Approximately 70 COVID-19 patients were given a combination of Linagliptin at 5mg per day with Insulin to study the changes in blood glucose levels, CRP levels and need for mechanical ventilation	Patients receiving Linagliptin in addition to insulin required ∼75% less assisted mechanical ventilation and ∼66% less death compared with only insulin receiving patients	[87,88] (NCT04542213)
	Approximately 89 non-diabetic COVID-19 patients were selected. One group received standard therapy while the other group received sitagliptin in addition to standard therapy and CBC, CRP, D-dimer, LDH and serum ferritin were measured	Sitagliptin with standard therapy improved clinical outcomes, radiological scores and inflammatory biomarkers in patients than standard therapy alone	[91]
	Approximately 112 diabetic COVID-19 patients were selected. One group received metformin + standard therapy while the other group received sitagliptin in addition to metformin + standard therapy and CRP, ferrin, procalcitonin, D-dimer, LDH were measured	Sitagliptin and metformin combination appeared to be more effective as compared with monotherapy of metformin	[92]

## Conclusion

Limited reports suggest that membrane-bound DPP-4 may interact with the SARS-CoV-2 spike glycoprotein, and thus may propagate viral infection. DPP-4, in addition to its disrupting role on incretin hormones, potentiates inflammatory conditions in multiple organs such as the lungs, heart and other target organs for SARS-CoV-2, causing fatal consequences. DPP-4 inhibitors may reduce inflammatory conditions, lessen severity of the disease and prevent organ damage. Recent clinical reports suggest DPP-4 inhibitors could be repurposed for treating SARS-CoV-2 infection for several reasons. By preventing inflammation through DPP-4 inhibition or through inhibition of TLR4 activation, DPP-4 inhibitors could reduce the production of cytokine storms and the level of CRP caused by SARS-CoV-2 infection. DPP-4 inhibitors may also reduce blood glucose levels and improve diabetogenic conditions, which appears to be a comorbidity in COVID-19. In both diabetic and non-diabetic subjects, DPP-4 inhibitors have been demonstrated to reduce blood pressure and hypertension, another common occurrence in COVID-19 patients. Thus, DPP4 inhibitors show potential to reduce the severity of COVID-19 disease and could be a potential choice for COVID-19 treatment in diabetic subjects.

## Future perspective

As DPP-4 inhibitors have multiple routes of reducing the severity of COVID-19, the use of different gliptins alone or in combination with other treatment strategies may become useful. A clearer understanding of how DPP-4 interacts with SARS-CoV-2 and facilitate the viruses entry into host cells, how sDPP-4 acts as decoy receptor to prevent viral entry and how use of different inhibitors of DPP-4 reduce the severity of the disease is required. Several randomized human trials are in progress for the use of DPP-4 inhibitors on COVID-19 patients. The results of these trials may provide a hope for the future use of DPP-4 inhibitors in combating this virus.

**Executive summary**

- Diabetic patients are more susceptible to COVID-19, with mortality rates up to three-times higher compared with non-diabetics.
- DPP-4 (CD26), which is a membrane-bound proteolytic enzyme, has been reported as a major entry receptor of MERS-CoV suggesting possible interaction with SARS-CoV-2 as well.

**DPP-4 & its implications in COVID-19**

- DPP-4 is present both as a membrane-bound form in most cell types and as a soluble form in the circulation.
- The lung is the most affected target organ for novel coronavirus and is rich in DPP-4 to promote inflammatory signals.
- COVID-19-infected patients with diabetes are reported to have reduced levels of soluble DPP-4.

**Virus-DPP-4 interaction**

- *In silico* studies suggest the interaction of the S1 subunit of SARS-CoV-2 spike glycoprotein with DPP-4.
- Soluble DPP-4 acts as a decoy receptor that, by binding the virus in circulation, prevents interaction with membrane DPP-4 and reduces entry into the host cells.

**DPP-4 inhibitors & COVID-19**

- DPP-4 inhibitors (also known as gliptins) are oral anti-diabetic drugs that seem to have a beneficial role in reducing the severity of COVID-19.
- COVID-19 patients with a maintained regime of gliptins required less intubation during hospitalization and reduced severity and mortality due to infection.

**Repurposing DPP-4 inhibitors in COVID-19 treatment**

- DPP-4 inhibitors antagonize the effects of SARS-CoV-2 by interfering with virus-host cell interaction, and therefore reduce inflammation and macrophage activity in the lungs.
- DPP-4 inhibitors also reduce COVID-19 associated comorbidities caused by increased blood pressures (SBP and DBP) leading to hypertension in both diabetic and non-diabetic subjects.
- In diabetic subjects, DPP-4 inhibitors upregulate soluble DPP-4 level in circulation, and deplete virus-host cell interaction.

**Dual potential of DPP-4 inhibitors against SARS-CoV-2 immunopathogenesis**

- RBD of SARS-CoV-2 spike glycoprotein interacts with TLR4 and induces inflammatory cascade in lungs.
- DPP-4 inhibitors impair TLR4 activation and protect the lungs from inflammatory injury.

**Randomized trials**

- Randomized trials provided evidence for the beneficial role of DPP-4 inhibitors in both diabetic and non-diabetic COVID-19 subjects.

**Conclusion**

- DPP-4 inhibitors interfere with SARS-CoV-2-host cell interaction, prevent inflammatory injury of the target organs, reduce blood pressure and hypertension that minimize the mortality rate of the COVID-19 infected subjects.

**Future perspective**

- As DPP-4 inhibitors reduce COVID-19 severity, its candidacy as a repurposed drug for controlling and treating the disease should gain considerable significance in the near future.
